# Emerging Approaches for Solid Tumor Treatment Using CAR-T Cell Therapy

**DOI:** 10.3390/ijms222212126

**Published:** 2021-11-09

**Authors:** Hyunmin Chung, Haiyoung Jung, Ji-Yoon Noh

**Affiliations:** 1 Immunotherapy Research Center, Korea Research Institute of Bioscience and Biotechnology (KRIBB), 125 Gwahak-ro, Daejeon 34141, Korea; hyunmin543@kribb.re.kr; 2 College of Pharmacy, Chungnam National University, Yuseong-gu, Daejeon 34134, Korea; 3 Department of Functional Genomics, Korea University of Science and Technology (UST), 113 Gwahak-ro, Yuseong-gu, Daejeon 34113, Korea

**Keywords:** CAR-T cell, solid tumor, challenge, combination therapy

## Abstract

Cancer immunotherapy is becoming more important in the clinical setting, especially for cancers resistant to conventional chemotherapy, including targeted therapy. Chimeric antigen receptor (CAR)-T cell therapy, which uses patient’s autologous T cells, combined with engineered T cell receptors, has shown remarkable results, with five US Food and Drug Administration (FDA) approvals to date. CAR-T cells have been very effective in hematologic malignancies, such as diffuse large B cell lymphoma (DLBCL), B cell acute lymphoblastic leukemia (B-ALL), and multiple myeloma (MM); however, its effectiveness in treating solid tumors has not been evaluated clearly. Therefore, many studies and clinical investigations are emerging to improve the CAR-T cell efficacy in solid tumors. The novel therapeutic approaches include modifying CARs in multiple ways or developing a combination therapy with immune checkpoint inhibitors and chemotherapies. In this review, we focus on the challenges and recent advancements in CAR-T cell therapy for solid tumors.

## 1. Introduction

Immunotherapy is becoming vital in treating cancer. Chimeric antigen receptor (CAR)-T cell therapy has shown remarkable efficacy in hematologic malignancies, including diffuse large B-cell lymphoma (DLBCL), B cell acute lymphoblastic leukemia (B-ALL), and multiple myeloma (MM), with five FDA approvals. Among immunotherapies, immune checkpoint inhibitors (ICIs) have also presented outstanding results, which might change the landscape of cancer immunotherapy in the future. Conventional chemotherapy affects the whole body, while the targeted therapies are more tissue specific. Therefore, they are limited to certain mutations and could confront novel mutations. Cancer immunotherapies retain tumor recognizing activity by using antibodies or CARs while promoting tumor cell lysis, through stimulation of cytotoxic immune cells. CAR-T cell may provide the best strategy as it possesses the advantages of immunotherapy. CAR-T cells can be used to repress refractory cancer cells after chemotherapy, irrespective of their underlying oncogenic driver mutations. Additionally, once CAR-T cells are sustained for a couple of years, they could potentially prevent the tumor relapse unless the tumor antigen changes.

The immune system plays a critical role in recognizing and eradicating foreign antigens, such as microbes or mutated cells, through immunosurveillance [1,2]. To exploit this homeostatic process through CAR-T cell therapy, understanding their mechanism of action is critical. First, CD8 or CD4 molecules (T cell receptor—TCR) on T cells recognize the non-peptide binding regions on the major histocompatibility complex proteins (MHC) class I or II molecule of the target cell or antigen-presenting cell (APC). Second, CD28 on T cell binds to either CD80 or CD86 on APCs, which leads to the production of effector cytokines by the APC [3]. Once the signals induce complete activation of T cells, the T cells can clonally expand to eradicate target cells. CARs are designed and reinforced to resemble TCR and co-stimulatory molecules that can detect tumor associated antigens (TAAs), resulting in clonal expansion and tumor eradication. The synthetic receptor CARs are expected to be efficient even if the MHC is downregulated in some tumors.

Based on previous clinical trials on hematologic malignancies, several important facts should be considered while developing novel CAR-T cell therapy. First, at least one co-stimulatory signaling domain in the CAR is necessary; second, differential expression of target antigen on tumor and normal tissue is required; third, tumor antigens that are targeted by CAR-T cells could be suppressed, which leads to therapeutic failure (i.e., CD19 antigen loss after anti-CD19 CAR-T cell exposure in hematologic malignancies); fourth, lymphodepletion prior to CAR-T cell infusion is considered to be necessary at present; finally, significant side effects can occur, including cytokine release syndrome (CRS) and neurotoxicity [4]. Additionally, limited tumor trafficking of infused CAR-T cells is one of the obstacles in solid tumor CAR-T cell therapy. Herein, we will describe the challenges in CAR-T cell therapy for solid tumor treatment in detail and focus on the recent preclinical and clinical studies, including combination therapies with CAR-T cells.

## 2. Tumor-Specific CAR-T Cell Immunotherapy

### 2.1. Hematologic Malignancies

Since the first FDA approval in 2017, there are five CAR-T cell therapies are available to date. They are tisagenlecleucel (tisa-cel, Novartis: refractory and relapsed (r/r) B-ALL, r/r DLBCL, CD19-41BB-CD3z CAR-T, cryopreserved PBMC), axicabtagene ciloleucel (axi-cel, Kite Pharma: r/r DLBCL, CD19-CD28-CD3z CAR T, fresh PBMC), brexucabtagene autoleucel (brex-cel, Kite Pharma: r/r mantle cell lymphoma, CD19-CD28-CD3z CAR T, enriched T cell), lisocabtagene maraleucel (liso-cel, Juno Therapeutics & Bristol Myers Squibb: r/r DLBCL, CD19-41BB-CD3z CAR T, 1:1 mixture of CD4:CD8 T cell), and idecabtagene vicleucel (ide-cel, Bristol Myers Squibb: r/r MM, BCMA-41BB-CD3z CAR T, CD8 T cell) [5]. CAR-T cells developed by Kite Pharma, axi-cel and brex-cel, consist of CD28 as a co-stimulatory domain, whereas, the rest of CAR-T cells contain 41BB. The CAR was transduced into fresh PBMC for axi-cel, while enriched T cell was used to generate brex-cel. Similarly, the latest CAR-T cells such as liso-cel and ide-cel are derived from isolated CD4 or CD8 T cells, which might have shown better efficacy and received FDA approvals.

Four out of five CAR-T that target CD19 exploit the mouse monoclonal antibody FMC63 for their single-chain variable fragment (scFv), which targets antigen specifically [6]. Thus, these CAR-T cell therapies have been approved for treating patients with CD19-positive B cell malignancies among non-Hodgkin’s lymphoma, such as r/r DLBCL, B-ALL, and mantle cell lymphoma [7,8]. Comparable to a clinically efficient scFv, FMC63—identifying a unique scFv that exhibits higher affinity, lesser “on-target, off-tumor” toxicity, and lesser tonic signaling of CARs on T cell—are important aspects of CAR-based adoptive cell transfer therapy. Multiple myeloma (MM) is the second most frequently diagnosed hematologic malignancy and the most common type of myeloma [9]. Several CAR-T cell therapies are being investigated for MM, and the most advanced one targets B cell maturation antigen (BCMA) [9]. Recently, BCMA CAR-T therapy showed outstanding results in heavily pretreated MM patients, leading to a FDA approval of idecabtagene vicleucel (ide-cel, Bristol-Myers Squibb) [10]. In addition, FDA granted priority review to ciltacabtageneautoleucel (cilta-cel, J&J, Legend biotech), a bi-valent nanobody-modulated CAR-T, which showed 80% stringent complete response (sCR) in the phase 2 study (NCT03548207) [11,12].

### 2.2. Solid Tumors

To date, approximately 200 clinical trials of CAR-T cells against solid tumors have been launched worldwide. CAR-T cell therapy is less effective in solid tumors than in hematologic malignancies [13]. One of the critical obstacles is to select an antigen that is exclusively expressed in tumor tissue. Nonetheless, results from preclinical and clinical studies continue to be reported. Solid tumor antigens that have been targeted the most by CAR-T cells are mesothelin (MSLN), ganglioside (GD2), glypican-3 (GPC3), human epidermal growth factor receptor 2 (HER2), carcinoembryonic antigen (CEA), epidermal growth factor receptor and its variants (EGFR/EGFRvIII), prostate-specific membrane antigen (PSMA), prostate stem cell antigen (PSCA), and claudin 18.2 [14]. In this section, we will briefly introduce examples of CAR-T cell studies on lung cancers and brain tumors. Lung cancer is the major cause of cancer-related deaths, and no treatment exists for certain types of brain tumor; therefore, CAR-T cell therapy could provide an attractive treatment option for both tumor types.

#### 2.2.1. Lung Cancer

Lung cancers are categorized into two histological types, non-small cell lung cancer (NSCLC) and small cell lung cancer (SCLC) [15,16]. SCLCs are rarer and typically more aggressive. NSCLCs can be classified into four subtypes as follows: lung adenocarcinoma (LUAD), lung squamous cell carcinoma (LUSC), large cell carcinoma, and bronchial carcinoid tumor. Driver mutations among female non-smokers are known as *KRAS*, *EGFR*, *BRAF*, and *ALK* fusions [16]. CAR-T cell therapy is being investigated in phase I and phase II clinical trials, mostly for NSCLCs. For example, PSCA CAR-T (NCT03198052), GPC3 CAR-T (NCT02876978), Mucin 1 (MUC1) CAR-T (NCT02587689), CEA CAR-T (NCT02349724), and MSLN CAR-T (NCT02414269) [17,18]. Recently, receptor tyrosine kinase-like orphan receptor 1-specific (ROR1-specific) CAR T cells were also evaluated preclinically [19,20]. ROR1 protein is highly expressed in some hematologic and epithelial cancer cells, including lung, breast, colon, pancreas, renal, and ovarian cancers [21], and presumably plays a role in malignant transformation [22]. It has been demonstrated that ROR1 expression is limited in normal adult tissues, suggesting that immunotherapies targeting ROR1 could be feasible [23]. Additionally, clinical trials of ROR1 CAR-T cells for NSCLC are ongoing (NCT02706392).

#### 2.2.2. Brain Tumor

Brain tumors are usually categorized by the tumor initiation site. Glioblastoma multiforme (GBM) is the most common brain cancer, which grows rapidly, is destructive, and represents high mortality. Neuroblastoma usually arises outside the central nervous system, while primary central nervous system lymphoma (PCNSL) is a tumor arising from the lymphatic tissue within the brain itself. Evaluation of CAR-T cell therapy on GBM has been intensively conducted, with promising results in preclinical models; however, a lack of efficacy has been reported in clinical trials with the CAR-T cell monotherapy [24]. TAAs have been identified and clinical trials are ongoing, as exampled by interleukin-13 receptor alpha 2 (IL13Rα2) (NCT02208362, NCT04003649) [25], HER2 (NCT03383978) [26], B7-H3 (NCT04077866), EGFRvIII (NCT02844062), and GD2 (NCT04099797) [27,28]. In addition, clinical trials on the FDA-approved CD19 CAR-T cells for PCNSL and secondary CNS lymphoma are ongoing (NCT04134117, NCT04608487, NCT03484702) [29]. It was demonstrated that CD19 CAR-T cell could indeed completely eradicate the tumor with a single dose, although this preclinical study was conducted by intracerebral injection [30].

## 3. Challenges of CAR-T Cell Therapy for Solid Tumors

The application of CAR-T cell therapy in treating solid tumors is restricted by (1) a limited array of targetable antigens, (2) heterogeneous antigen expression in solid tumors, (3) CAR structure, (4) challenges in CAR-T cell production, (5) limited T-cell fitness, (6) inefficient homing and infiltration to tumor tissue, and (7) the immunosuppressive tumor microenvironment (TME) [4]. In addition, adverse events induced by CAR-T cells, including CRS and neurotoxicity, critically endanger the patients and are sometimes fatal. Thus, novel therapeutic strategies that use lower CAR-T cell doses with highly efficient CAR and combination therapies are urgently needed. In this section, we describe the challenges of CAR-T cell therapy for solid tumors due to the T cell intrinsic and extrinsic factors.

### 3.1. T Cell Intrinsic Factors

#### 3.1.1. T Cell Exhaustion

Continuous receptor-mediated stimulation of effector T cells can trigger further differentiation of the T cells into the exhausted state, which results in reduced T cell persistence and increased expression of inhibitory receptors [31,32]. Interestingly, modification of CD28 co-stimulatory domain with reduced activity has shown to improve CAR-T cell function in vivo [33]. The roles of CD28 and 41BB are extensively investigated as co-stimulatory domains of CAR. It has been demonstrated that CD28 signaling promotes glycolytic metabolism and effector memory phenotype; whereas, 41BB signaling can promote oxidative phosphorylation and a central memory phenotype [34]. The third-generation CAR with the combination of CD28 and 41BB represented more robust proliferation after infusion than the second-generation CAR using only CD28 [35,36]. Nonetheless, it has been reported that the conventional CAR construct could not be potentiated efficiently by using two intracellular signaling domains [37,38], and a severe CRS has been reported after the third generation CAR-T cell treatment [39].

The density of CARs on T cells or the subset of T cells could also affect the efficacy and toxicity. It has been demonstrated that immunological synapse between CAR-T cells and target cells is correlated with CAR-T cell function [40,41]. Interestingly, the high affinity of CAR-T cells to target antigens enables recognition at a low density, but it can also increase the risk of off-tumor toxicities. Also, affinity exceeding a threshold (*K*_d_ < 10^−8^ M) had no difference in inducing T cell activity [42,43], suggesting that higher antigen affinity does not necessarily improve the efficacy of CAR-T cell therapy. Furthermore, the biological source for CAR-T cell therapy can vary, as they are derived from autologous T cells. This can affect the therapeutic strategy. For instance, a clinical trial with JCAR017 (liso-cel, FDA approved for r/r DLBCL) has been recommended to use a fixed ratio of CD8 to CD4 cells for B cell lymphoma (NCT04245839) [44].

#### 3.1.2. Toxicity of CAR-T Cells

The adverse events of CAR-T cell therapy can be divided into CRS, immune effector cell-associated neurotoxicity syndrome (ICANS), on-target and off-tumor effects of CAR-T cells, cardiotoxicity, and hypersensitivity reactions [45,46]. CRS is defined as an excessive release of cytokines, such as interleukin (IL)-1, IL-6, interferon (IFN)-γ, and IL-10, by infused immune cells or the body’s innate immune cells. Severe CRS is caused by multi-organ failure, coagulopathy, and respiratory system damage, and can be life threatening [47]. ICANS is also observed in patients treated with CAR-T cell therapy at about 0–50% incidence, and symptoms include hallucinations, encephalopathy, seizures, and cerebral edema [48]. These adverse events can be relieved by monoclonal antibodies against the IL-6 receptor, IL-1 receptor antagonist [49], or targeting granulocyte–macrophage colony-stimulating factor (GM-CSF) [50] (tocilizumab, anakinra, or lenzilumab, respectively). Tocilizumab was approved for treatment of CRS, while anakinra and lenzilumab have shown the therapeutic efficacy for both CRS and neurotoxicity. However, methods for assessing and predicting potential toxicities before administration remain elusive.

### 3.2. T Cell Extrinsic Factors

#### 3.2.1. Immunosuppressive Tumor Microenvironments (TMEs)

Suppressive immune cells in the TMEs, such as regulatory T (Treg) cells, myeloid-derived suppressor cells (MDSC), and tumor-associated macrophages (TAM), secrete cytokines blocking CAR-T cell function significantly [51,52]. Cytotoxic T cell functions are impaired by IL-4, IL-10, and transforming growth factor (TGF)-β. Moreover, TGF-β and IL-10 recruit MDSCs and Tregs, which can prohibit cytotoxicity of effector T cells against tumor cells [53,54].

Besides suppressive immune cells and cytokines, the TME also prohibits the trafficking and infiltration of CAR-T cells from peripheral blood. T cell migration to tumor lesions is hindered in solid tumors due to a physical barrier built by extracellular matrix or mismatching chemokine receptors of immune cells and tumor-derived chemokines [55], which is not the case in lymphoid tumors. Chemokines secreted in the TME, such as CXCL12, have been shown to inhibit CAR-T cell migration into the solid tumor [56,57]. Endothelin B receptors on the endothelium of blood vessels within the tumor or other extracellular matrix proteins are involved in the disruption of T cell adhesion and migration [58]. Other factors, such as low pH and hypoxic conditions, are also harmful for T cells to persist [59,60]. Because cancer cells are highly proliferative and have high amounts of glycolysis and glutaminolysis [61], various metabolites are accumulated in the TME. Among them, indoleamine-2,3-dioxygenase (IDO) is known to suppress T cell responses [62,63].

Ligands of immune checkpoints such as programmed death-1 ligand (PD-L1/L2), expressed on tumor cells, cause inhibition of the effector T cell function [64]. In normal conditions, immune checkpoints are critical for maintenance of self-tolerance and avoiding overreaction-induced tissue damage of the T cell response. Once the ligands bind to immune checkpoints such as PD-1 and CTLA-4, the CD28-mediated PI3K-Akt signaling pathway is prevented, which reduces T cell activation, proliferation, and survival [65,66].

#### 3.2.2. Tumor-Associated Antigens

Tumor antigens can be divided into two groups: TAAs and tumor-specific antigens (TSAs). The former includes CD19, ERBB2, and p53, which are enriched in cancer cells but are also expressed on normal tissues [67]. TAAs could provide a target for CAR-T cell therapy [68,69]. Some of TSAs are neoantigens present on MHC molecules [70,71], which are unique to the patients rather than to the tumor [72]. Thus, neoantigens are suitable targets for personalized cancer vaccines and not for general CAR-T cell therapy [73]. They are also often downregulated in tumors as a result of the immune-escaping mechanism of tumors [74,75].

“On-target, off-tumor toxicity” often limits the development of CAR-T cell therapies for solid tumors, as most of the TAAs, though overexpressed on tumor cells, are not tumor-specific, posing severe threats [39]. For instance, in a clinical trial with carbonic anhydrase IX (CAIX) CAR-T cells for treating metastatic renal cell carcinoma, an unexpected grade 2–4 hepatotoxicity after transfusion was observed. It was discovered that CAIX CAR-T cells attack CAIX-positive bile duct epithelial cells [76]. About 33 types of CARs are already currently being investigated for solid tumors [27,44]. To avoid “on-target, off-tumor” toxicity, efficient TAAs should be identified for successful CAR-T cell therapy in solid tumors [59,77,78]. More importantly, markers or methods that can predict the toxicity over normal tissue by CAR-T cells need to be considered.

Heterogeneous antigen expression is a hallmark of cancer and another reason for the failure of CAR-T cell therapy in solid tumor [79]. In addition, multiple clinical trials have demonstrated that the loss of antigen after CAR-T cell immunotherapy has led to significant tumor relapse in patients with ALL and glioblastoma (NCT01626495, NCT01029366, and NCT02208362) [80,81,82,83]. Taken together, identifying TAAs that are only upregulated in tumor tissue and not in the normal tissues is a critical step for the development of CAR-T cell therapy. Targeting multiple antigens to lower the dose of CAR-T cells and compensate for the antigen loss in vivo can be additional strategies to improve CAR-T cell therapy.

## 4. Emerging Approaches for the Improvement of CAR-T Cell Therapy

**Figure 1:**
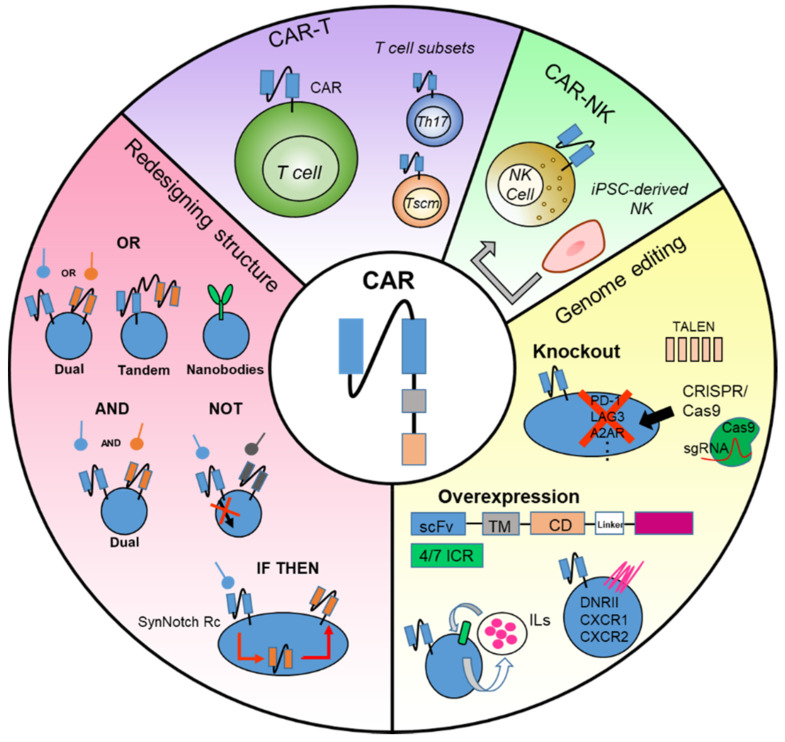
Summary of novel approaches for employing chimeric antigen receptors (CARs). CAR construct has been advanced and redesigned to express a variety of combinations, including diverse scFvs and intracellular signaling domains. CARs can be expressed in either T cells, T cell subsets, or NK cells to develop anti-tumor immune cell and gene therapies. Additional gene engineering has been conducted in immune cells to enhance the CAR-T cell persistence and anti-tumor efficacy. *Th17*—T helper 17; *Tscm*—stem cell-like memory T cell; *iPSC*—induced Pluripotent stem cell; *NK*—natural killer; *TALEN*—transcription activator-like effector nucleases; *CRISPR*—clustered regularly interspaced short palindromic repeats; *sgRNA*—single guide RNA; *PD-1*—programmed cell death-1; *LAG3*—lymphocyte activation gene 3; *A2AR*—A2A adenosine receptor; *DNRII*—TGF-β dominant negative receptor II; *scFv*—single chain variable fragment; *TM*—transmembrane; *CD*—cytoplasmic domain; *ICR*—inverted cytokine receptor; *ILs*—interleukins.

**Figure 2:**
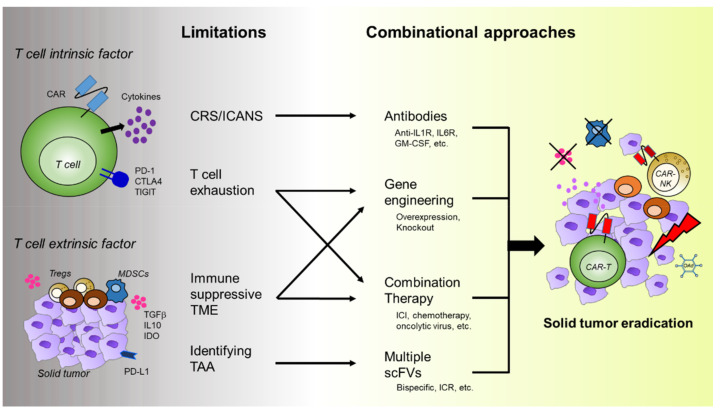
Therapeutic strategies to overcome current limitations and improve CAR-T cell efficacy in solid tumors. The current problems of CAR-T cell therapies in solid tumors can be categorized into T cell intrinsic factor and T cell extrinsic factor, which is described in Section 3. The conventional CAR-T cells can be further modified genetically to express more CARs, impair inhibitory immune checkpoints (such as PD-1 and CTLA-4), or retain activation genes, such as chemokine receptors and interleukins. In addition, a variety of drugs including immune checkpoint inhibitors are currently being tested in combination with CAR-T cell preclinically and clinically. Alternatively, cancer vaccine or CAR-NK cells are also being developed for the next generation cancer immunotherapy. *CAR*—chimeric antigen receptor; *ICI*—immune checkpoint inhibitor; *PD-1*—programmed cell death-1; *CTLA4*—cytotoxic T lymphocyte-associated protein 4; *TIGIT*—T cell immunoreceptor with Ig and ITIM domains; *CRS*—cytokine release syndrome; *ICANS*—immune effector cell-associated neurotoxicity syndrome; *GM-CSF*—granuolocyte-macrophage colony-stimulating factor; *Treg*—regulatory T cell; *MDSC*—myeloid-derived suppressor cell; *TGF-β* —transforming growth factor beta; *IDO*—indoleamine 2,3-dioxygenase; *TME*—tumor microenvironment; *TAA*—tumor-associated antigen; *ICI*—immune checkpoint inhibitor; *ICR*—inverted cytokine receptor; *OAd*—oncolytic adenovirus.

A summary of novel approaches for employing CARs and the strategies of combination therapies for the improvement of CAR-T cell efficacy in solid tumors are depicted in Figure 1 and Figure 2, respectively. The current status of the strategies throughout preclinical to clinical studies is summarized in Table 1.

### 4.1. Redesigning CAR Structure

The CAR consists of several domains, including scFv, hinge, transmembrane (TM), co-stimulatory domain 1 or 2, and signaling domain. Notably, while an scFv primarily provides target specificity, CAR can be further optimized in the hinge or TM sequences depending on the epitope location [84,85]. In addition, CAR-T cell activity can be modulated by intracellular domains [86], although the excessive activation of receptor signaling could result in lesser efficacy of CAR-T cell therapy [31,87]. Nonetheless, one of the most critical components of CAR structure is the scFv [88]. Researchers have designed CAR-T cells with two or more scFvs targeting distinct antigens [89,90]. Multiple CARs are operated in either “OR”, “AND”, or “NOT” logical switches. Among them, only the “OR” logic gate has been studied clinically, while “AND” or “NOT” strategies seem to be better options for solid tumors [44,91]. In this section, we will introduce recent studies employing multiple CARs to improve CAR-T cell efficacy as well as to overcome “on-target, off-tumor” adverse events.

#### 4.1.1. Multiple CARs with “OR” Strategy

Immunotherapies with multiple CARs could be a mixture of two CAR-T cell lines, co-expression of two to three separate CARs in individual T cells (dual CAR, triple CAR), or tandem CARs. Pooled CAR-T cells, such as targeting HER2/IL-13Rα2 for glioblastoma and CD19/CD123 for B-ALL, exhibited higher levels of cytokine release than the individual CAR-T cell lines, suggesting that the strategy might increase the efficacy of CAR-T cells [92,93]. However, simultaneous infusion of two CAR-T cell lines could lead to the escape of both antigens and imbalance in each CAR-T cell populations. In addition, dual CAR-T cells revealed better efficacy than pooled CAR-T cells in B-ALL animal model [93], though the clinical evidence is limited at the moment. Studies with dual CAR-T cells containing distinct CAR molecules with anti-HER2 scFv-CD28-CD3z (HER2 scFv-CD28-CD3z) and IL13Rα2 scFv-CD28-CD3z, or CD19 scFv-41BB-CD3z and CD123 scFv-41BB-CD3z, or CD19 scFv-OX40-CD3z and CD22 scFv-41BB-CD3z showed enhanced anti-tumor efficacy [94,95]. Phosphorylation of zeta-chain-associated protein kinase 70 (ZAP-70) was detected in high levels with the infusion of dual CAR-T cells, indicating that enhanced downstream signal transduction can be achieved using two CARs simultaneously [92,93,96].

Tandem CAR-T cells have also been investigated, including CD19 and HER2 scFv-CD28-CD3z, HER2 and IL13Rα2 scFv-CD28-CD3z, and CD19 and CD20 scFv-CD28-CD3z [97,98,99]. Advantageously, the CAR-T cells could preserve anti-tumor activity even after one of the antigens escaped. One of the limitations of this is the viral vector package size due to the extended scFvs. Recently, nanobodies, heavy chain antibodies found naturally in sharks and camelids, have been discussed as substituents of scFvs [100,101]. Nanobodies are composed of 110–130 amino acids, lack light chains, and present weak immunogenicity [102,103]. The feasibility of expressing nanobody-based tandem CAR-T cells has been studied with targeting CD20 and HER2 [104].

#### 4.1.2. Multiple CARs with “AND” or “NOT” Strategy

It has been suggested that “AND” or “NOT”-gate scFvs may allow the eradication of tumors more precisely than “OR”-gate strategy, which would alleviate on-target, off-tumor effects [105]. The combination of “AND” and “NOT” strategies can be diverse. Basically, the CAR-T cell will only be activated by both antigens, and the effect of the CAR-T cell will be prohibited by the second antigen, respectively. As an example, CARs of mesothelin scFv-CD3z and folate receptor alpha (FRα) scFv-CD28 have been introduced simultaneously, resulting in an improved tumor trafficking and reduction of toxicity [106].

The “NOT” system can be obtained by including one CAR with full activation domains and another CAR with inhibitory domains, which recognize TAA and normal tissue target, respectively. In dual CAR-expressing strategy, one CAR consisted of CD19 scFv-CD28-CD3z; whereas, another CAR was designed to contain an inhibitory intracellular signaling domain from PD-1 or CTLA-4, specific to an antigen present in normal tissue, prostate-specific membrane antigen (PSMA) [107]. Notably, the dual CAR-T cells transduced with both CD19 scFv-CD28-CD3z and PSMA scFv-PD-1-intracellular trail could be inactivated by PSMA, suggesting that this concept might be feasible for optimizing the on-target, off-tumor effect of CAR.

#### 4.1.3. Multiple CARs with “IF/THEN” Strategy

The advancement of CAR design strategy has led to the development of brilliant systems to improve CAR-T cell efficacy for solid tumors that exploit the “IF/THEN” logic gate. For example, a customizable CAR using a synthetic Notch (synNotch) receptor system has been investigated, where the first CAR interaction induces transcription of the second CAR through the synNotch receptor [108,109]. Since the expression of antigens in “IF/THEN” logic gate is not as strict as “AND” gate, it could still be effective in the context of antigen loss.

### 4.2. Genome Editing of CAR-T Cell

#### 4.2.1. Knockout of Inhibitory Molecules

Since genome editing technologies, such as CRISPR-Cas9 and TALEN, are available for human peripheral blood-derived T cells, researchers have knocked out T cell inhibitory molecules, such as PD-1, lymphocyte activation gene 3 protein (LAG-3), and adenosine A2A receptor (A2AR), and reported enhanced CAR-T cell anti-tumor efficacy in xenograft models in vivo [110,111,112]. Among them, PD-1 knockout (KO) CAR-T cells using CRISPR-Cas9 RNA and protein (RNP) technology has recently been in clinical trials (NCT03081715, NCT02867332, NCT02867345, NCT02793856, NCT03044743), and it has been demonstrated that PD-1 KO T cells or CAR-T cells are safe and feasible for patients with solid tumors [113,114,115]. In addition, a combined genome engineering strategy on T cell receptor (TCR)/β-2 microglobulin/PD-1, followed by CAR expression to generate universal CAR-T cells, showed effective CAR-T cell function in vivo [116]. Recently, another combined genome engineered T cells (KO of TCRα, TCRβ, and PD-1) with overexpression of cancer-specific TCR transgene (NY-ESO-1) were transferred to patients with advanced, refractory cancer, and it has been observed that this strategy is feasible (NCT03399448) [117]. Similarly to PD-1, LAG-3 can suppress T cell proliferation and activation through blockade of intracellular signal transduction [66]. It has been demonstrated that LAG-3 KO CAR-T cells displayed robust anti-tumor activity in the murine xenograft model [111]. Similarly, CRISPR-Cas9-mediated deletion of A2AR has enhanced CAR-T cell activities with scFvs against HER2 or Lewis Y in animal models [112].

NR4A are a family of orphan nuclear receptors, composed of NR4A1, NR4A2, and NR4A3, and are known to work as transcription factors [118]. They are upregulated during CD8 T cell exhaustion, induced by constitutively active NFAT that could not interact with an adjacent AP-1 [119]. Thus, it has been suggested that NR4A maintains the accessibility of “exhaustion-related” regions that bind NFAT without AP-1. Triple KO of Nr4a in tumor-infiltrating lymphocytes (TILs) displayed potent effector functions as follows: decreased inhibitory receptor expression, increased cytokine production, and strong enrichment in chromatin binding motifs of transcription factors involved in effector function [120]. The authors showed that treatment of tumor-bearing mice with CAR-T cells lacking all three NR4A transcription factors resulted in tumor regression and prolonged survival.

Tyrosine-protein phosphatase non-receptor type 2 (PTPN2) is an intracellular enzyme that can modulate downstream signaling of T cell receptors. PTPN2-deficient T cells noticeably repressed tumor formation in mice, and KO of PTPN2 in HER2 CAR-T cells enhanced tumor eradication in a preclinical model with HER2 positive mammary tumors [121]. Similarly, KO of diacylglycerol kinase (DGK), a potential inhibitory immune checkpoint, has promoted solid tumor regression by synergized EGFRvIII CAR-T cells in vivo [122].

#### 4.2.2. Overexpression of T Cell Stimulus Genes

CARs are usually employed to promote cytotoxic T cell function, and CARs are usually delivered by lentiviral transduction. Like CAR, additional genes can be overexpressed to activate the CAR-T cells further, such as the dominant negative mutation of TGF-β receptor type II (DNRII), chemokine receptors, and transcription factors [123,124]. DNRII-expressing immune cells can avoid inhibitory TGF-β effect and DNRII-modified PSMA CAR-T cells have exhibited increased persistence and activity in vivo xenograft model. These are now in phase I clinical trial (NCT03089203) [125]. Similar to the approach to delete NR4A to downregulate the T cell exhaustion-related NFAT signaling [120], overexpression of AP-1 factor c-Jun can activate CAR-T cells by promoting exhaustion resistance [126].

Chemokine receptor-modified CAR-T cells showed enhanced tumor trafficking and efficacy [127]. Recently, CXCR2-CAR-T cells were generated to target hepatocellular carcinoma, in which the T cell infiltration, as well as anti-tumor effects, were improved [128]. Preclinical studies with CD70-CXCR1 or CD70-CXCR2 CAR-T cells have revealed that the T cells were markedly enhanced to migrate and survive in intra-tumoral region, such as GBM, resulting in complete tumor regressions. CD70 and IL-8, a ligand for CXCR1 or CXCR2, are upregulated in GBM [129].

#### 4.2.3. Promoting Inflammatory and Proliferative Activities of CAR-T Cells

Interleukines (ILs) promote T cell proliferation and release of inflammatory cytokines. ILs can also reverse immunosuppressive TMEs, which may be advantageous for treating solid tumors. Thus, novel IL secreting systems or inverted cytokine receptor (ICR) have been incorporated in CAR-T cells, which was reviewed in Zhang et al. [130]. CAR-T cells targeting prostate stem cell antigen (PSCA) were further engineered to improve T cell persistence and expansion, via co-expression of TGF-β receptor-41BB and IL-4 receptor-IL7 receptor signaling domain (also called 4/7 ICR) [131]. Similarly, 4/21 ICR was shown to activate STAT3 pathway, leading to T helper 17 (Th17)-like polarization of the CAR-T cells and enhanced persistence in vivo [132]. Interestingly, Th17 and CD8+ T 17 (Tc17) were used to express CAR in the murine breast tumor model, which has shown to enhance CAR-T cell persistence and trafficking in tumor in combination with STING (Stimulator of Interferon Genes) agonist and PD-1 blockade [133].

Clinical trials of CAR-T cells co-expressed with cytokines such as IL-7, CCL19, IL-15, and IL-21 for solid tumors are under way [134,135,136]. Recently, Lange et al. reported that a chimeric GM-CSF/IL-18 cytokine receptor (GM18) that links CAR-T cell activation to MyD88 signaling pathway could promote CAR-T cell persistence, which resulted in potent antitumor efficacy in a preclinical study with solid tumors [137]. Another study showed potentiation of CAR-T cell activity induced by T cells engineered to secrete the FLT3 ligand that can promote epitope spreading [138].

### 4.3. Combination Therapy

#### 4.3.1. Chemotherapy or Other Strategies

Lymph-depleting chemotherapy, prior to CAR-T cell infusion, is considered a necessary procedure at present, which enables expansion and persistence of the CAR-T cells as well as reduces regulatory T cells and myeloid cells [139]. Previously, patients with nine hematologic malignancies (mostly CLL) who had received cyclophosphamide prior to CD19 CAR-T cell infusion represented increased CAR-T cell persistence [140].

As described above, neurotoxicity is one of the major adverse events of CAR-T cell therapy, and GM-CSF is a key factor. Neutralization of GM-CSF had indeed improved CD19 CAR-T cell efficacy in a patient-derived xenograft model, which led to a phase 2 clinical trial of anti-GM-CSF in combination with CAR-T cell therapy [50]. Lenzilumab (Humanigen), an anti-human GM-CSF monoclonal antibody, was administered with axi-cel in adults with r/r DLBCL in the multicenter ZUMA-19 study. Recent reports show that the combination induced an 83% overall response rate (ORR) (*n* = 5), including four complete responses. Evaluation of the safety and efficacy of lenzilumab, with all commercially available CD19 CAR-T cell therapies, is being planned. Another study for reducing CAR-T cell toxicity has been performed using dasatinib, an oral tyrosine kinase inhibitor, which interferes with the lymphocyte-specific protein tyrosine kinase (LCK) and thereby inhibits CAR activation-mediated CD3z signaling [141]. In this study, the authors tested CD19 CAR-T cells and demonstrated that dasatinib could induce the “function-off” phase, and the overall therapeutic outcome and benefit of CAR-T cell therapy were similar upon removal of dasatinib in an animal model of CRS [142], which had been reported separately.

To promote CAR-T cell activity against solid tumors, combinations with drugs targeting TMEs are feasible. One of the most targeted molecules of TMEs is TGF-β1, and many studies have reported the development of TGF-β1 inhibitors [143]. Galunisertib is an oral TGF-β1 inhibitor, and its combination with CD133 CAR-T cells or HER2 CAR-T cells has been reported to be effective against solid tumors in animal models [144]. It has also been reported that MDSCs and Treg cells in TME can be repressed by all-trans retinoic acid [145,146], vitamin D3 derivative, and anti-CD25, OX40, and CCR4 antibodies, respectively [13]. Future studies may provide novel strategies to inhibit MDSCs or Treg cells in combination with CAR-T cell therapy.

Recently, a combination of cell carrier-delivered oncolytic adenovirus (OAd) and CAR-T cells was reported to be effective against lung tumors in animal models [147]. The adenovirus was engineered to encode IL-12 and anti-PD-L1 antibodies, and mesenchymal stem cells (MSCs) were used as cell carrier, delivering and producing OAd and eradicating tumor cells. Additionally, HER2 CAR-T cells were administered to eliminate lung cancer cells in vivo. The oncolytic herpes virus that expresses human GM-CSF has achieved FDA approval for the treatment of melanoma [148], and the delivery of OAd with MSCs has also been tested [149]. Thus, this strategy is now open for clinical trials (NCT 03740256).

#### 4.3.2. Immune Checkpoint Blockade

Once CAR-T cells penetrate to the immunogenically silent solid tumors, immune checkpoint inhibitors may reverse CAR-T cell exhaustion and maintain the function [150,151]. Thus, redesign of CARs involves in addition of anti-PD-1 blocking scFv to the TAA targeting CARs [152,153]. Rafiq et al. developed CD19 CAR-T cells that could secrete PD-1 scFv upon CD19 CAR activation in the tumor site, showing anti-tumor efficacy [154]. The idea for redesigning CARs to improve efficacy varies, as described above. For instance, PD-1 scFv-CD28 receptor was expressed with CAR, and the CAR-T cell increased persistence as well as anti-tumor efficacy in mesothelioma and prostate cancer xenograft models [154,155]. Another study demonstrated that dominant negative PD-1 receptors without intracellular signaling domains could prevent the inhibitory effect of PD-L1 and PD-L2 on tumor cells and lead to enhanced survival in mesothelioma and lung tumor mouse models [156]. Clinical trials on the effect of PD-1 scFv, in combination with CD19 CAR or EGFR CAR, for treating multiple advanced tumors, have been launched (NCT03030001, NCT03182803, NCT03182816, NCT02873390).

Since immune checkpoint inhibitors (ICIs), such as ipilimumab (anti-CTLA-4 antibody), nivolumab, and pembrolizumab (both anti-PD-1 antibodies) achieved FDA approval, the landscape of immunotherapy for treating various solid tumors in recent years has changed [157]. Most clinical trials with ICIs in combination with cell therapy are combining tumor-infiltrating lymphocytes (TILs, e.g., lifileucel) [158], with one of the ICIs for treating melanoma, NSCLC, or head and neck cancer (NCT02027935, NCT03296137, NCT03638375, NCT03645928, NCT01993719). The clinical trials for treating solid tumors using CAR-T cells in combination with PD-1 blockade have been scheduled [159,160] and reviewed in [161]. The patient with DLBCL received autologous CD19 CAR-T cells, and after 26 days, pembrolizumab was administered. By day 45, the percentage of T cells expressing PD-1/EOMES decreased, and expansion of CAR-T cells was observed with the regression of multiple lesions [162]. Another study was reported with r/r neuroblastoma patients, while the addition of pembrolizumab did not significantly improve the anti-tumor efficacy of CAR-T cells, as opposed to the combination with cyclophosphamide and fluodarabine [163]. Recently, the patients with malignant pleural mesothelioma were treated with MSLN CAR-T cells in combination with anti-PD-1 agents, which showed encouraging outcomes [164].

### 4.4. Development of CAR-NK Cell Therapy

Natural killer cells (NK cells) have been utilized in anti-cancer immunotherapy, especially against hematologic tumors [165,166]. NK cells contain both activation receptors and inhibitory receptors and rapidly act against tumor cells without prior sensitization. Reduction of inhibitory receptor–ligand interaction can also initiate the NK cell anti-tumor activity, indicating that malignant cells, often with suppressed MHC class I molecule expression, may prove a target for NK cells. Upon activation, NK cells release granzyme and perforin to lyse target cells in a similar fashion to cytotoxic T cells. Thus, infusion of NK cells or CAR-NK cells has an advantage over CAR-T cell therapy and has demonstrated overall safety in clinical trials, even in the allogeneic haploidentical transplant [167,168].

CARs can be transduced in NK cells, and the use of a variety of CAR-NK cells has been demonstrated in in vitro, preclinical, and clinical studies. Like CAR-T cell development, novel targets or combination therapies using CAR-NK cells are being investigated. CAR-NK cells expressing the activating receptor NKG2D could recognize MDSCs and eliminate these immunosuppressive immune cells in the TME [169,170]. Furthermore, it has been shown in the mouse model that CAR-NK cells modify the TMEs by secreting proinflammatory cytokines and chemokines, promoting the infiltration of CAR-T cells [169]. Nevertheless, tumor infiltration of infused CAR-NK cells is also limited. To improve it, NKG2D CAR-NK cells were infused in combination with a CD73 antibody that suppresses adenosine production in TMEs, resulting in an enhanced anti-tumor efficacy [171]. Research on overexpressing the chemokine receptor, such as CXCR1, has also been conducted for improved tumor infiltration of CAR-NK cells [172].

The feasible allogeneic setting of NK cell therapy has allowed researchers to expand NK cell sources, which includes peripheral blood (PB)-NK, cord blood (CB)-NK, immortalized cell lines like NK-92 cells, and induced pluripotent stem cell (iPSC)-derived NK cells [173,174]. CB-NK and iPSC-derived NK cells could provide “off-the-shelf” therapy, as CB-NK cells could recover after cryopreservation, while iPSCs can be expanded to larger scale, reviewed in [175]. Besides producing sufficient amount of NK cells, iPSC-NK cells have also known as its equal or greater cytotoxicity against cancer cell lines, compared to PB-NK and NK-92 cells [176,177]. Expandable iPSC-NK cells can be easily genetically modified to knockout inhibitory receptors or to express non-cleavable mutated CD16 receptor, and CARs [178]. Recently, iPSC-NK cells were demonstrated to enhance inflammatory cytokine production and tumor lysis via recruiting T cells in cooperation with anti-PD-1 antibody [179]. Furthermore, the first clinical investigation of Fate Therapeutics’ off-the-shelf iPSC-NK product, FT500 is underway for treating advanced solid tumors in combination with checkpoint inhibitors (NCT03841110).

To date, more than 100 clinical trials of NK cell-based cancer immunotherapy are listed. It includes the umbilical cord blood hematopoietic stem cell-derived NK cells, at the allogeneic partial HLA-matched setting, designated as an orphan drug by FDA for AML. Notably, a phase I/II clinical trial for treating relapsed or refractory CD19-positive hematologic malignancy using CB-derived CAR NK cells, and seven out of eleven patients showed complete remission, with no significant CRS or ICANS (NCT03056339) [180]. Besides the direct killing, NK cells can induce tumor cell lysis through antibody-dependent cellular cytotoxicity (ADCC). Thus, ex vivo-expanded human peripheral blood NK cells can be infused either in the form of monotherapy or in combination with monoclonal antibodies for treating advanced solid tumors (NCT03319459). In this study, CHIR99021, a GSK3 kinase inhibitor, was used to generate the expanded NK cells, as it could promote the anti-tumor effector functions and enhance the ADCC effect of NK cells [181]. Further phase I or II clinical trials are ongoing, such as combining NK cells with nimotuzumab (anti-EGFR antibody) for treating late-stage malignancies (NCT03554889) and NK cell infusion for advanced malignancies following multi-line therapies (NCT03619954). For CAR-NK cells, clinical trials to evaluate the safety and efficacy of HER2 CAR-NK cell (NCT04319757) and MUC1 CAR-NK cell (NCT02839954) [182], etc., are emerging.

## 5. Conclusions

Combination therapies with immunotherapies and chemotherapies are being developed extensively to obtain meaningful outcomes in clinical trials, especially for solid tumors with no response to the conventional therapies. Technical advancements in genome editing, viral vector construction, and immune cell isolation and expansion have made the novel CAR-T cell therapies more feasible. In this review, we described the challenges of CAR-T cell therapies, with a focus on the emerging combination therapeutic strategies and future directions. It could be a complicated decision for the patients to choose between CAR-T cell therapy or other adoptive cell therapies, such as NK cell, TIL, or CAR-NK cell therapy. More importantly, it requires biomarkers for adverse events like the “on-target, off-tumor” effect. Therefore, potential toxicities like CRS should be considered when planning treatments. Nevertheless, we believe that it is worthwhile because once it works, these living drugs can recognize and further eradicate the remaining cancer cells. With an appropriate combination therapy, CAR-T cells could work much better in the body, with increased persistence, reduced exhaustion, and better tumor trafficking. In conclusion, more progression in CAR-T cell therapy and increased understanding of cancer immunology could lead to development of novel therapies and help patients with advanced solid tumors.

## Figures and Tables

**Table 1:** Summary of novel strategies of CAR-T cell therapy described in Section 4, in combination with other CARs, genetically engineered T cells, chemotherapy, and immune checkpoint inhibitors.

Target	CAR-T Cell Mode †	Combination (Strategy)	Malignancy	Preclinical Study	Clinical Trial	Clinical Trial Register	Ref
CD19/CD22	41BB	Bispecific CAR-T cell	Leukemia/Lymphoma	-	-	NCT03233854	[89]
CD19/CD20	41BB	Bispecific CAR-T cell	Leukemia/Lymphoma	-	-	NCT03019055	[97]
HER2/IL3Ra2	CD28	Pooled CAR-T cells	Glioblastoma model	-			[92]
CD19/CD123	41BB	Pooled CAR-T cell	Leukemia model	-			[93]
PSMA/CD19	PD1/CD28	Bispecific CAR-T cell	Leukemia model	-			[107]
CD22/CD19	41BB	Tandem CAR-T cell	B-ALL	-	-		[94]
CD19/MSLN	synNotch/41BB	Bispecific CAR-T cell (synNotch CAR)	Leukemia model	-			[108]
CD19/CD22	OX40/41BB	Bispecific CAR-T & Pembrolizumab	DLBCL	-	-	NCT03287817	[95]
CD20/HER2	CD28	Nanobody based tandem CAR-T cell	Leukemia cell			*	[104]
EPHA2 or HER2/GM-CSF	CD28/IL18R	IL18R (MYD88 signaling)	Ewing sarcoma model Osteosarcoma model	-			[137]
GD2	41BB/IL7Ra	C7R (STAT5 signaling)	GD2-expressing brain tumors, RR neuroblastoma, other GD2-expressing cancers	-	-	NCT04099797 NCT03635632	[135]
PSCA/TGF-β /IL4	CD28/41BB/IL7Ra	4/7 ICR	Pancreatic adenocarcinoma model	-			[131]
GPC3/IL4	CD28/IL21R	4/21 ICR	IL-4-expressing hepatocarcinoma model	-			[132]
GPC3	41BB	IL15, IL21, iCasp9	Liver cancer Rhabdomyosarcoma	-	-	NCT04715191	[136]
N/A	N/A	Knockout of PD1	Esophageal cancer Renal cell carcinoma Castration resistant prostate cancer Metastatic NSCLC Advanced stage EBV	- - - - -	- - - - -	NCT03081715 NCT02867332 NCT02867345 NCT02793856 NCT03044743	
CD19	CD28	Knockout of NR4A	Lymphoma model, Melanoma model	-			[120]
CD19	41BB	Knockout of TCR/β2 microglobulin/PD1	ALL model	-			[116]
HER2	CD28	Knockout of A2AR	Ovarian carcinoma model	-			[112]
HER2	CD28	Knockout of PTPN2	Breast cancer model	-			[121]
EGFR	41BB	Knockout of DGK	Glioblastoma model	-			[122]
HER2	41BB	Overexpression ofc-Jun	Osteosarcoma model	-			[126]
GPC3	41BB	Overexpression ofCXCR2	Hepatocellular carcinoma model	-			[128]
CD70	41BB	Overexpression of CXCR1 or CXCR2	Glioblastoma model Pancreatic tumor model Ovarian tumor model	-			[129]
PSMA	41BB	Overexpression of TGF-β DNRII	Prostate cancer	-	-	NCT03089203	[125]
CD19	CD28 (Yescarta^®^)	Lenzilumab	DLBCL	-	-	ZUMA-19	[50]
CD19	41BB	Dasatinib	Lymphoma	-			[141]
CD133/HER2	CD28, 41BB	Galunisertib	Glioblastoma, Breast cancer cell			*	[144]
HER2	CD28	Cell-carrier-delivered oncolytic adenovirus	NSCLC	-	-	NCT03740256	[147]
EGFR	41BB	Anti-PD1 scFv secretion	Lung gastric liver cancer	-	-	NCT02862028	[152,154]
MSLN	41BB	Anti-PD1 Ab expression	Refractory prostate cancer	-	-	NCT03030001	[153]
MSLN	41BB	Anti-CTLA4/PD1 Ab expression	Non-hematologic malignancies	-	-	NCT03182803	[159]
EGFR	41BB	Anti-CTLA4/PD1 Ab expression	NSCLC	-	-	NCT03182816	[160]
CD19	41BB	Pembrolizumab	DLBCL	-	-	NCT02650999	[161]
CD19	41BB	Pembrolizumab	NHL	-	-	NCT02030834	[162]
GD2	CD28,OX40	Pembrolizumab	Neuroblastoma	-	-	NCT01822652	[163]
CD19	41BB	alemtuzumab	B-ALL	-	-	NCT02808442 NCT02746952	[96]
N/A	TIL	Ipilimumab	Metastatic melanoma	-	-	NCT02027935 NCT03296137	[158]
N/A	TIL	Nivolumab	Metastatic melanoma	-	-	NCT03638375	
N/A	TIL	Nivolumab, Pembrolizumab	Metastatic melanoma	-	-	NCT03645928	

N/A—not applicable; B-ALL—acute B lymphoblastic leukemia; NSCLC—non-small-cell lung cancer; DLBCL—diffuse large B-cell lymphoma; NHL—non-Hodgkin’s lymphoma; HER2—human epidermal growth factor receptor 2; PSMA—prostate-specific membrane antigen; MSLN—mesothelin; PSCA—prostate stem cell antigen; TGF—transforming growth factor; EPHA2—ephrin type A receptor 2; IL18R—interleukin 18 receptor; GM-CSF—granulocyte–macrophage colony-stimulating factor; MYD88—myeloid differentiation factor 88; C7R—constitutively active IL7 receptor; RR—refractory and relapsed; ICR—inverted cytokine receptor; iCasp9—inducible caspase-9; EGFR—epidermal growth factor receptor; GPC3—glypican 3; PD1—programmed cell death protein 1; A2AR—adenosine A2A receptor; PTPN2—protein tyrosine phosphatase non-receptor type 2; DGK—diacylglycerol kinases; DNR—dominant negative receptor; CTLA4—cytotoxic T lymphocyte antigen 4; NR4A—nuclear receptor subfamily 4A; CXCR—CXC chemokine receptors. † CAR-T cell mode means the signaling domain used in the CAR construct. * In vitro analysis only.
